# AISOA-SSformer: An Effective Image Segmentation Method for Rice Leaf Disease Based on the Transformer Architecture

**DOI:** 10.34133/plantphenomics.0218

**Published:** 2024-08-05

**Authors:** Weisi Dai, Wenke Zhu, Guoxiong Zhou, Genhua Liu, Jiaxin Xu, Hongliang Zhou, Yahui Hu, Zewei Liu, Jinyang Li, Liujun Li

**Affiliations:** ^1^ Faculty of Electronic Information and Physics, Central South University of Forestry and Technology, Changsha, 410004 Hunan, China.; ^2^ College of Bangor, Central South University of Forestry and Technology, Changsha, 410004 Hunan, China.; ^3^ Plant Protection Institute, Hunan Academy of Agricultural Sciences, Changsha, 410125 Hunan, China.; ^4^ Department of Soil and Water Systems, University of Idaho, Moscow, ID 83844, USA.

## Abstract

Rice leaf diseases have an important impact on modern farming, threatening crop health and yield. Accurate semantic segmentation techniques are crucial for segmenting diseased leaf parts and assisting farmers in disease identification. However, the diversity of rice growing environments and the complexity of leaf diseases pose challenges. To address these issues, this study introduces an innovative semantic segmentation algorithm for rice leaf pests and diseases based on the Transformer architecture AISOA-SSformer. First, it features the sparse global-update perceptron for real-time parameter updating, enhancing model stability and accuracy in learning irregular leaf features. Second, the salient feature attention mechanism is introduced to separate and reorganize features using the spatial reconstruction module (SRM) and channel reconstruction module (CRM), focusing on salient feature extraction and reducing background interference. Additionally, the annealing-integrated sparrow optimization algorithm fine-tunes the sparrow algorithm, gradually reducing the stochastic search amplitude to minimize loss. This enhances the model’s adaptability and robustness, particularly against fuzzy edge features. The experimental results show that AISOA-SSformer achieves an 83.1% MIoU, an 80.3% Dice coefficient, and a 76.5% recall on a homemade dataset, with a model size of only 14.71 million parameters. Compared with other popular algorithms, it demonstrates greater accuracy in rice leaf disease segmentation. This method effectively improves segmentation, providing valuable insights for modern plantation management. The data and code used in this study will be open sourced at https://github.com/ZhouGuoXiong/Rice-Leaf-Disease-Segmentation-Dataset-Code.

## Introduction

Rice is one of the most important food crops in the world [1–3], but its production is affected by various leaf diseases. Rice leaf diseases cover a wide range of symptoms caused by pathogens such as fungi, bacteria, and viruses, which include spots or blotches of different shapes, colors, and sizes on rice leaves. These diseases pose a potential threat to rice health and yield [4]. Therefore, it is crucial to accurately segment and identify leaf disease species and take timely control measures. However, traditional manual methods suffer from the disadvantages of a high workload, low efficiency, and susceptibility to fatigue. With the development of computer technology, the practical application of semantic segmentation technology has become a development trend in modern rice cultivation.

Rice leaf pest and disease segmentation is the core technology for automatic disease detection and identification and provides a reliable basis for rice leaf detection systems by accurately dividing diseased leaf images [5]. In recent years, image segmentation technology based on deep learning [6], which can accurately identify and localize affected areas [7–9], has become key in this field, helping farmers take timely control measures and effectively curb the spread of disease. This not only provides strong technical support for accurate and efficient disease management but also brings new vitality to agricultural production.

In general, deep learning-based semantic segmentation networks can be categorized into 2 types: unsupervised learning, represented by clustering-based networks (SegSort; [10]) and generative model-based networks (GANs; [11]), and supervised learning, represented by encoder–decoder structures (UNet; [12]) and Transformer [13]. Unsupervised learning [14] does not require labeled data and allows the model to autonomously learn the structure, patterns, and relationships of the data for segmentation tasks. However, unsupervised learning lacks a clear goal and is sensitive to initial conditions and hyperparameters, thus leading to less stable segmentation results. For diseased rice leaf segmentation, unsupervised learning cannot guarantee that the segmented region has agricultural significance, nor can it effectively address the occlusion, overlap, and deformation of rice, thus making it difficult to meet the needs of precision agriculture. Unlike unsupervised learning, supervised learning requires a large amount of manually labeled data with clear task objectives and usually achieves high prediction performances because the models can learn effective feature relationships from labeled data.

In recent years, supervised learning methods have been increasingly applied to the detection and segmentation of rice leaf diseases [15–17], providing references for our research, and the relevant literature is shown in Table 1. Of the 3 previous studies listed in Table 1, each method has certain shortcomings, including a lack of flexibility in dealing with irregular and diverse disease features due to the fixed weight parameter settings of the Squeeze-and-Excitation (SE) module, limitations in dealing with complex backgrounds due to the use of simple scale transformations, and the need for a large number of iterations in the parameter space, which leads to poor performance in fuzzy boundary segmentation. Given these weaknesses, it is reasonable to select Segformer [18], which is based on the Transformer architecture, as the baseline network for experimentation. Segformer has a strong global modeling capability and can capture long-distance dependencies, making it especially suitable for handling complex disease features. However, Segformer also has certain limitations, including difficulties in capturing irregular rice leaf diseases due to the single linear layer in the multilayer perceptron (MLP). Moreover, although the Segformer architecture excels in capturing global dependencies, it requires the design of attention components that can better consider spatial relationships to effectively distinguish between rice leaf disease areas and complex backgrounds, and the optimizer can be improved to enhance the model’s ability to recognize features of fuzzy boundaries. By improving Segformer, we aim to achieve more precise segmentation in the task of rice leaf disease segmentation while further exploring the interaction between disease features and module structures, thus improving the model’s effectiveness in segmenting rice leaf diseases.

**Table 1.** Documentation for research to identify plant leaf diseases

Research	Methodology	Fields	Advantages	Drawbacks
Chen et al. [51]	BLSNet	Rice leaf disease	The integrated multiscale extraction function improves the accuracy of lesion segmentation.	The SE module lacks flexibility for highly irregular and diverse leaf diseases. Additionally, the dataset only includes 109 cases of rice bacterial leaf streak, resulting in weak model generalization.
Feng et al. [52]	DFFANet	Rice leaf disease	DCABlock and FFM can extract the deep and shallow features of rice as much as possible.	It uses a continuous 3 × 3 convolution for feature extraction, which ignores complex spatial relationships and lacks flexibility. In addition, the simple scale transformation has difficulty dealing with the complex background of rice diseased leaf photos.
Putra et al. [53]	UNet with incorporated randomized search	Rice leaf disease	Stochastic search can be explored over a wide range of parameter spaces, which would enhance the accuracy and efficiency of the model in identifying and segmenting rice leaf diseases.	Random search may be inefficient in large parameter spaces with many optimized parameters, potentially missing combinations that better handle fuzzy regions.

**Fig. 1.**
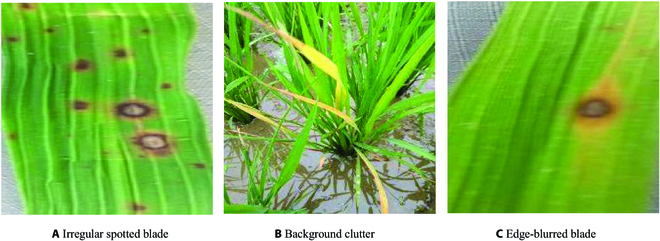
Three problems in rice segmentation.

As shown in Fig. 1, there are 3 main challenges in the current rice leaf disease segmentation process: (a) Irregularly speckled diseased leaves in rice leaf disease images often have complex textures and shapes. This complexity makes accurate recognition and separation challenging for segmentation models, thereby affecting their accuracy. (b) Cluttered background elements, such as other plants, soil, or weeds, can interfere with the model’s judgment. This interference may lead to misidentification of background noise or irrelevant elements as diseased regions, causing false segmentations. (c) The edge blurring problem in diseased images significantly impacts the performance of the segmentation network. Sometimes, this issue can result in errors in the segmentation results, such as incorrectly labeling healthy leaf regions as diseased.

To solve the problem of irregular speckled leaves in plant diseases, Lu et al. [19] proposed the Transformer model (GeT) combined with phantom convolution, which generates feature maps through a convolutional backbone network and learns semantic features in depth with the Transformer encoder to achieve accurate segmentations. However, its fixed parameter update strategy easily leads to poor model generalizability.

To solve the problem of complex backgrounds in plant disease photos, Li et al. [20], on the other hand, used an FWDGAN-based approach to identify tomato leaf diseases coupled with a depth-separable convolutional discriminator network (DSC-Discriminator), which combines deep and global features and improves image quality through depth-separable convolutions. However, this network mainly analyzes the disease image in its overall dimension and is prone to lack flexibility when faced with multiple interfering factors.

To solve the problem of blurred edges in disease images, Liu et al. [21] proposed a self-supervised pretraining method based on Transformer so that the former model has more obvious discriminative feature representations of plant diseases and thus achieves accurate filtering of the background interference elements of the disease and pest images. However, conventional optimization algorithms tend to trap model training in local optimums, resulting in the inability to adapt to or recognize new disease data patterns and anomalies. These studies provide valuable insights and directions for our rice leaf pest and disease segmentation model, and through more in-depth innovation and research, we expect to achieve greater breakthroughs and progress in the field of rice leaf pest and disease segmentation.

The contributions of this paper are as follows:

1. For the semantic segmentation network to obtain rich features of rice leaf diseases, we constructed an accurately labeled dataset containing Tungro and Brown Spot diseases. All the disease regions in the dataset were labeled with Labelme software, and labeled maps were generated.

2. To address the above issues, we propose a new model called AISOA-SSformer that integrates 3 innovative improvement points to enhance the performance and stability of the model. First, the model introduces a linear embedding layer called sparse global-update perceptron (SGUP), which combines the exponential moving average (EMA) and weighted moving average (WMA) methods to dynamically adjust the weights and update the parameters using a fixed sliding window to respond more efficiently to critical changes and to ensure model stability when recognizing and identifying irregular disease characteristics. Second, we developed a novel attention salient feature attention mechanism (SFAM) to improve the quality of feature maps through the spatial reconstruction module (SRM) and the channel reconstruction module (CRM). SRM is mainly used to filter important features and reduce background noise, whereas the CRM optimizes channel information and continuously focuses on key features so that the model can more effectively distinguish rice leaf disease regions from complex backgrounds. Finally, we introduce a new optimization algorithm, the annealing-integrated sparrow optimization algorithm (AISOA), which combines the stochastic exploration property of the sparrow algorithm and the stepwise constraints of the annealing mechanism to help the model avoid falling into local optima while enhancing the recognition of fuzzy boundary features and maintaining the stability of the training process and the robustness of the model. The combination of these 3 techniques provides strong support for enhancing the performance of the model in complex application scenarios.

3. The Segformer-based AISOA-SSformer proposed in this paper achieved an 83.1% mean intersection over union (MIoU) and an 80.3% Dice coefficient on the self-constructed dataset. This method can be used to effectively extract rice leaf disease features with complex backgrounds and irregular shapes. Rice leaf diseases with fuzzy edges can be distinguished and segmented effectively. Overall, the method is able to accurately detect rice leaf diseases and provides a reference for disease control in mass rice production.

## Datasets and Methodology

### Data acquisition

**Fig. 2.**
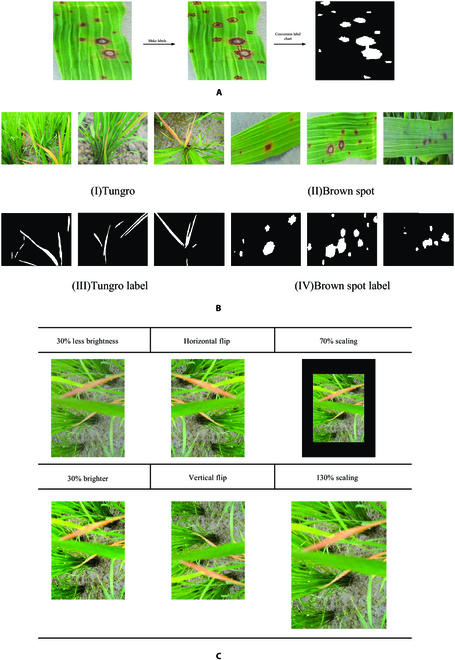
(A to C) Examples of the labeling process and data enhancement of labels and original images.

Rice pest and disease segmentation datasets are the basis of this research. We chose 2 typical rice leaf spot types, brown spot and rice Tungro, to detect spots at different disease stages to avoid the segmentation effects being weak because of insufficient study of each disease stage. Brown spot disease results in the formation of round or ovoid spots on rice leaves approximately 2 to 5 mm in diameter with reddish brown margins and yellowish brown centers, sometimes with black dots in the middle. The brown spots are usually distributed in the upper part of rice leaves, and the middle and lower parts of the leaves are less infested. After infection with the Tungro virus, rice plants exhibit symptoms such as dwarfing, leaf curling, leaf coloration, and orange or reddish coloration of the leaf tips. We selected the rice leaf spot dataset from PlantVillage in a natural environment containing 2 rice leaf pest datasets, which provided enough training data for the semantic segmentation algorithm. We selected the images with obvious leaf spot characteristics for the next step in the collected images, which totaled 2,005 images, including a total of 1,005 images for Tungro disease and 1,000 images for brown spot disease. To facilitate training, we used Labelme as the data annotation software. With the help of a team of agricultural experts, we labeled the lesions in all the images into categories and accurately selected all the pixels in the lesion area. In the process of labeling the 2 diseases, we uniformly named the label mask “label,” used Labelme to label and obtain the corresponding JSON file, and obtained a total of 4,794 labels for 1,000 sheets of brown spot disease and a total of 4,350 labels for 1,005 sheets of Tungro disease through a statistical analysis. Finally, the images were cropped to a uniform size of 256 × 256 pixels. Figure 2 shows the annotation process (Fig. 2A) and an example of the original and annotated images used for training on 2 rice leaf diseases (Fig. 2B).

### Data preprocessing

Since the training of deep neural network models requires diverse images to extract effective features, enhancement of the original dataset is needed to increase data diversity and improve model generalizability. The data enhancements in this paper includes (a) applying photometric distortion, such as light and dark adjustments to the luminance, to simulate sunlight changes. (b) Perspective transformation enhancements, such as mirror flipping of images. (c) Scale transformation enhancements, such as the scaling of images. To minimize the impact of the data distribution on network training, the number of enhancements for the Tungro and Brown Spot categories is balanced. Taking Tungro as an example, the data enhancement results are shown in Fig. 2C. The annotations for each image are stored in PNG format.

### AISOA-SSformer

**Fig. 3.**
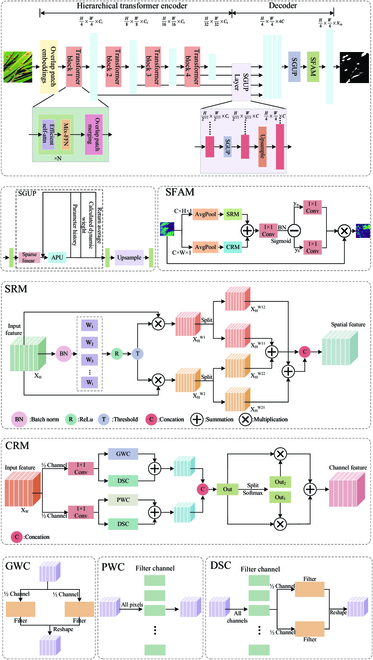
AISOA-SSformer structure diagram.

Considering the characteristics of rice leaf diseases, this paper proposes AISOA-SSformer, a rice leaf disease segmentation method based on Segformer_b0, whose structure is shown in Fig. 3A. AISOA-SSformer consists of 2 parts: (a) a hierarchical Transformer decoder that generates high-resolution coarse features and low-resolution fine features, and (b) a lightweight SGUP layer decoder that fuses these multilevel features to produce the final semantic segmentation mask. First, we propose the SGUP, which replaces the regular MLP in the original decoder. The constructed adaptive parameter updater (APU) is used to update the model training parameters in a more refined way, improving the stability and continuity of the network. On the optimizer, we propose the AISOA, which helps the model jump out of the local optimal point by mimicking the sparrow random exploration. At the tail of the SGUP layer decoder, this paper proposes the SFAM. This structure provides a rich and fine-grained feature representation for the rice semantic segmentation task by decomposing the feature map into horizontal and vertical directions and performing SRM and CRM operations on the feature maps in the 2 directions, respectively, which ensures that the model focuses on important features.

#### Sparse global-update perceptron

Rice leaf diseases usually involve many irregular speckled leaves, such as brown spots on brown spots, which are easily confused with the texture and color features of the rice itself, making it difficult for the segmentation algorithm to accurately distinguish them. In Segformer, the MLP [22] learns and extracts features at different levels through multilayer nonlinear transformations, but due to its single linear layer and simple parameter updating strategy, it is more sensitive to fluctuations and noise in the training data, which restricts its ability to handle complex data. To solve this problem, Wang and Zhang [23] proposed an improved deep learning method based on the EMA [24], which improves the stability and generalization ability of the network by using the EMA of the parameters during the training process. Based on the above research, this paper proposes the SGUP module for irregular spots in rice leaf diseases. The SGUP module enhances the MLP by considering the number of parameters in the training process. It also integrates the APU. Through dynamic weight adjustments and a new parameter update mechanism, the SGUP module captures and retains multiple disease regions. This reduces the possibility of missegmentation and improves segmentation accuracy. The structure of the SGUP is shown in Fig. 3B, which describes the operational process of the SGUP in detail.

To enhance the model’s robustness to irregular patches and improve segmentation accuracy, we propose an innovative method called the SGUP. This method replaces the traditional MLP in the network and is specifically designed to process complex spatial data such as irregular patches. First, the method preprocesses the input tensor *x* through dimensional rearrangement and flattening operations to convert the multidimensional data into one-dimensional vectors for subsequent linear processing. Then, the output tensor *y* is obtained by inputting a sparse linear layer (SparseLinear). The weight matrix is sparsified by zeroing some elements, which reduces the number of model parameters and improves training efficiency. This helps the model focus on key features and enhances its learning ability for highly irregular data patterns. Additionally, during forward propagation, we integrate the APU mechanism to optimize model parameters by combining the EMA and WMA [25] strategies.

The mechanism is designed with a sliding window of length “*n*” to track the history of the neural network model’s parameters. A larger value of “*n*” means that it utilizes a longer history of information, and more historical parameters are taken into account when calculating the weighted average, resulting in smoother parameter updates, fewer fluctuations, and a more stable model during the training process. In contrast, when setting a smaller value of “*n*,” the APU will only take into account the most recent historical parameters, which makes the parameter update more sensitive to recent changes, enabling the model to respond to parameter changes more quickly, but also tends to introduce more noise. For irregular patches in complex backgrounds, larger values of “*n*” provide more stable parameter updates, helping the model to remain stable in the face of complex and variable data, thus reducing the interference caused by background changes. For simpler tasks, smaller “*n*” values allow the model to adapt to data changes faster but also tend to introduce more noise and interfere with model training. Therefore, the choice of the “*n*” value needs to be weighed against the complexity of the task and the need for the model to respond to data changes.

We designed the SGUP to reflect the latest learning trend of the model and enhance its stability. Therefore, this approach provides significant performance improvements when dealing with data with complex spatial relationships, such as irregular patches. The forward propagation formulation and WMA calculation formula for the sparse linear layer are as follows:

$$y=x\times {\left(\text{weight}⊙\text{mask}\right)}^{T}+b$$ (1)

$$P_{W}=\frac{\sum_{l=1}^{n}\omega_{l}\cdot P_{l}}{\sum_{l=1}^{n}\omega_{l}}$$ (2)

The ⊙ in Eq. 1 denotes elementwise multiplication, where the mask is a matrix consisting of randomly generated sparsity masks that, when multiplied by the weight matrix weight, will result in a fraction of the elements being zero. *ω_l_* in Eq. 2 represents the weight of the *l*th parameter, *P_l_* is the value of the *l*th parameter, and *P_W_* represents the weighted average parameter.

Finally, the computed weighted average parameters are used to update the parameters of the EMA model. This smooths the parameter updating process by blending the preupdate parameters with the computed weighted average parameters and updating the model parameters with a fixed decay factor. The instability caused by random fluctuations in the data (e.g., the disturbance of irregular speckled blades) is effectively reduced, and the robustness of the model is improved by fully considering the neighboring parameters. The updating equations of the EMA are as follows:

$$P_{E}=P{'}_{E}+\left(1-\text{decay}\right)\cdot \frac{\sum_{l=1}^{n}\omega_{l}\cdot P_{l}}{\sum_{l=1}^{n}\omega_{1}}$$ (3)

where *P_E_* is the current EMA parameter, $P_{W}^{\prime }$ is the EMA parameter from the previous step, *ω_i_* is the weight of the parameter, which determines the importance of the parameter in calculating the new EMA parameter, and *P_i_* is the weight of the model.

We replace the MLP in the lightweight MLP layer and the MLP in the last layer of the decoder with the SGUP to optimize the decoder in segmenting the diseased rice leaves, where the optimized decoder operates as follows.

First, the decoder processes the rice leaf disease data through the hierarchical Transformer encoder. The original image is segmented into multiple overlapping patches using the overlap patch embedding module. Each patch is then mapped into a high-dimensional feature space through a convolution operation to extract local features from the image. These embedding vectors are then processed through a series of Transformer blocks, each of which contains the self-attention mechanism [26], Mix-FFN (a hybrid feedforward network), and patch embedding modules, which further process the disease features to capture the global dependencies. Finally, the multilayer feature *F_i_* is obtained through 4 block processes, and the 4 feature maps are upsampled to the same size through resizing. Each feature is individually passed through LayerNorm to unify the channel dimensions, followed by an upsampling operation to resize the features to ^1^/_4_ of their original size before concatenating them. Then, the spliced features *F* are fused again after 4 SGUPs, and finally, a separate SGUP is used to predict the segmentation mask *M*. This mask utilizes the spliced and fused features with a resolution of *H*/4 × *W*/4 × *N*, where *N* is the number of categories. This step directly generates the semantic segmentation output from the fused features, avoiding complex postprocessing or additional components. The above decoder operation process is represented by the following equation:

$${\overset{\cdot }{F}}_{i}=\text{SparseLinear}\left(C_{\varepsilon },c\right)\left(F_{\varepsilon }\right),\forall_{i}$$ (4)

$${\overset{\cdot }{F}}_{i}=\text{Upsample}\left(\frac{H}{4}\times \frac{W}{4}\right)\left({\overset{\cdot }{F}}_{i}\right),\forall_{i}$$ (5)

$$F_{i}=\text{SparseLinear}\left(4c,c\right)\left(\text{Concat}\left({\overset{\cdot }{F}}_{i}\right)\right),\forall_{i}$$ (6)

$$M=\text{SparseLinear}\left(C,N\right)\left(F\right)$$ (7)

In the equation, M is the prediction mask; SparseLinear(*C*_in_, *C*_out_)(·) represents the sparse linear layer operation with *C*_in_ and *C*_out_ as the input and output vector dimensions, respectively; and *F_i_* is the multilayer feature.

After the above operation, we utilize the lightweight MLP proposed by Segformer. In this structure, we replace the traditional MLP, which has only a single linear layer, with SparseLinear. The sparsity of SparseLinear means that there are many zeros in the weight matrices of the layers. This helps the model to better learn the sparsely distributed features of the data while maintaining computational efficiency. In this way, the model can focus more on those features that are more critical for recognizing irregularly shaped spots.

In addition, this mechanism captures rich multidimensional information in each forward propagation of the model by integrating the APU mechanism during each forward propagation of the SGUP, and real-time adaptation to changes in the input data is achieved by continuously and dynamically adjusting the model parameters. This continuous parameter adaptive updating strategy greatly enhances the model’s adaptive capability, especially when dealing with data with a high degree of irregularity and complexity, such as irregular disease spots on rice leaves. The experimental results show that the SGUP structure exhibits excellent performance when dealing with data with a high degree of variability, which enables the model to better understand and adapt to different data distributions and structural changes. Experiments on the SGUP are described in the “Effectiveness of SGUP” section.

#### Salient feature attention mechanism

As mentioned in the previous section, there are problems such as cluttered background elements in rice leaf pest images, which have a significant impact on the training of the deep learning model, such as increasing the difficulty of training the model and reducing its robustness. While the attention mechanism is widely used in image processing [27–32], the early SE [33] adaptively learns the weights of each channel by increasing the attention in the channel dimension and then assigns different weights to each feature according to the importance of each weight. This not only improves the performance of the network but also shows good robustness. Coordinate attention (CA) [34], which has emerged in recent years, targets the length and width of an image separately and better captures the global context information and long-range dependencies. Based on the above study, we propose a new attention mechanism, the SFAM, to achieve accurate rejection of the cluttered background elements through more efficient processing and reconstruction of input features and accurate capture of the spatial and channel relationships. The structure of the SFAM is shown in Fig. 3C, and it operates as follows.

First, we perform preliminary processing of the input feature maps, and we borrow the core idea from CA and Scconv [35]. First, the input rice disease feature maps are spatially dimensionally separated by 2 parallel avgpools and separated into 2 sets of features of C × W × 1 and C × H × 1, which are input into SRM and CRM for processing.

1. After inputting feature *X_H_* ∈ *N* × *C* × *H* × 1 into SRM, the processing steps are as follows. For the input rice feature map *X_H_* ∈ *N* × *C* × *H* × 1, we achieve better feature fusions by performing separation and reconstruction operations. A bulk normalization layer (BN) is first constructed. It normalizes the input feature *X_w_* by subtracting the mean *μ* divided by the variance σ to generate the intermediate value *X*_out_. This is followed by mapping *X*_out_ through a sigmoid function to generate a weight value *W* ranging between (0,1). This weight value is necessary for the subsequent gating operation, which is based on a certain threshold value to determine which features are important. Through this process, the model learns to distinguish between features that are more important and those that are less important for identifying leaf disease areas. Two new weight values, *W*_1_ and *W*_2_, are distinguished by comparing and judging *W* with the set gating threshold, gate_threshold, with *W*_1_ being the weight for the most informative rice leaf disease, i.e., the important feature, and *W*_2_ being the least informative rice leaf disease, i.e., the unimportant feature. Eventually, we select a more reasonable reconstruction strategy by multiplying the 2 weights *W*_1_ and *W*_2_ with the original feature *X_H_*, respectively, based on the different amounts of information in these 2 parts. This strategy combines information-rich features with less informative features to generate richer features as a way to optimize space utilization efficiency. To perform this reconstruction operation efficiently, we perform a further split operation on these 2 parts of the features to generate 4 different subfeatures $X_{H}^{W_{11}}$, $X_{H}^{W_{12}}$, $X_{H}^{W_{21}}$, and $X_{H}^{W_{22}}$, which are then cross-reconstructed to generate the new output $X_{H}^{W}$ combining both the informative and the uninformative parts, and the entire separation and reconstruction process can be expressed as follows:

$$X_{\text{out}}=\gamma \left(\frac{X-\mu_{B}}{\sqrt{\sigma_{B}^{2}+\in }}\right)+\beta$$ (8)

$$W=\text{Gate}\left(\text{sigmoid}\left(\mathrm{BN}\left(X_{\text{out}}\right)\right)\right)$$ (9)

$$\left\{\begin{array}{c} X_{H}^{W_{1}}=X_{H}⊗W_{1}\\ X_{H}^{W_{2}}=X_{H}⊗W_{2}\\ X_{H}=\left(X_{H}^{W_{11}}⊕X_{H}^{W_{22}}\right)⊕\left(X_{H}^{W_{21}}⊕X_{H}^{W_{12}}\right) \end{array}\right.$$ (10)

where ⊕ is the summation and ⊗ is the multiplication. After passing the pooled features through SRM, the important and unimportant features of rice leaf disease are distinguished. This allows the network to focus more on the important features related to rice identification and segmentation while ignoring irrelevant and cluttered background information. The improvement in feature extraction capability caused by this separation processing operation of the features is especially important when dealing with complex agricultural scenarios. The structure of SRM is shown in Fig. 3D.

2. The feature *X_H_* ∈ *N* × *C* × *W* × 1 is input into the CRM, which adopts the segmentation–transformation–compression–fusion strategy. To process the upper and lower features of rice separately and achieve a more reasonable allocation of computational resources, we divided the input rice leaf disease feature map into 2 parts with the same number of channels. Assuming C = channel, the ^1^/_2_ channel is input into the upper feature (UP) and the other ^1^/_2_ channel is input into the lower feature (LOW). Next, we compress the channels of the upper feature and the lower feature using 2 parallel 1 × 1 Conv layers to improve computational efficiency. This process results in obtaining 2 features, $X_{\mathrm{UP}}^{W}$ and $X_{\mathrm{LOW}}^{W}$. Among them, the upper feature can capture a wider range of contextual information, such as the overall shape of the leaf, while the lower feature focuses more on capturing the details of the disease on the leaf, such as the spots and the discolored areas. For the upper and lower features, we use more efficient convolutions, such as groupwise convolutions (GWC; [36]), pointwise convolutions (PWCs), and depthwise separable convolutions (DSC; [37]), instead of complex *k* × *k* convolutions, reducing the computational cost, which is schematically shown in Fig. 3F to H. DSC divides the convolution operation into 2 steps, depth convolution and PWC, which require only 1/channel subcomputation of the original convolution and are more computationally efficient. The PWC compensates for the convolution process with information loss and helps the information flow between the feature channels. GWC performs a grouping operation on the channels and uses a separate set of convolution kernels for each group to improve the overall efficiency. We perform a 3 × 3 GWC and DSC operation for the upper layer feature $X_{\mathrm{UP}}^{W}$, which aims to deeply extract the global and local feature information of rice disease at a low computational cost to obtain the upper layer feature map $Y_{1}^{W}$. On the other branch, we subject $X_{\mathrm{LOW}}^{W}$ to a DSC and a cheaper 1 × 1 PWC to extract the features with shallow hidden details of the disease site, and the generated lower feature map $Y_{2}^{W}\;$serves as a complement to the upper features. The operation of the 2 branches is represented as follows:

$$\left\{\begin{array}{c} Y_{1}^{W}=G\left(X_{\mathrm{UP}}^{W}\right)+D\left(X_{\mathrm{UP}}^{W}\right)\\ Y_{2}^{W}=P\left(X_{\mathrm{LOW}}^{W}\right)+D\left(X_{\mathrm{LOW}}^{W}\right) \end{array}\right.$$ (11)

Finally, the upper feature map $Y_{1}^{W}\;$and the lower feature map $Y_{2}^{W}$ are spliced in the channel dimension to obtain the fused feature map. Then, the Softmax operation is performed on the fused feature map to generate the attention weights. Next, the attention weight tensor is split into 2 layers in the channel dimension, which are used to further reorganize the features, and then are multiplied by the fusion feature map. Finally, the results of the 2 multiplications are summed to obtain the final feature map *Y_W_*.

Then, the $X_{H}^{W}$ and *Y_W_* generated by SRM and CRM are spliced in the channel dimension to obtain *y*, which is then passed through a 1 × 1 convolution layer, BN layer, and sigmoid activation function to perform the downscaling and feature transformation. To process the features in the length and width directions separately and generate more detailed attention weights, we split them into *y_h_* and *y_w_* in spatial dimensions. We then apply 2 convolution operations in parallel to convert them into an attention map. After passing the attention map through the sigmoid function, we multiply it by the main element identity of the original feature map. This yields the final feature map, and the operation flow after generating $X_{H}^{W}$ and *Y_W_* is shown as follows:

$$Y=\text{Concat}\left(X_{H},Y_{W}\right)$$ (12)

$$y=\text{sigmoid}\left(\mathrm{BN}\left(1\times 1\text{Conv}\left(Y\right)\right)\right)$$ (13)

$$\left\{\begin{array}{l} a_{h}=\text{sigmoid}\left(1\times 1\text{Conv}\left(y\right)\right)\\ a=\text{sigmoid}\left(1\times 1\text{Conv}\left(y\right)\right) \end{array}\right.$$ (14)

$$\text{Out}=\text{identity}\times a_{w}\times a_{h}$$ (15)

By integrating the spatial information modeling capability of SFAMs, this method significantly enhances the ability to capture the features of rice leaf diseases. SRM differentiates between information-dense and sparse areas, while CRM refines the feature processing and fusion, collectively improving the model’s sensitivity and recognition capability for foreground features. Additionally, the attention weights generated by 2 parallel convolutional layers and an efficient activation function, when combined with the original feature map, not only preserve important foreground feature information but also effectively suppress background noise and irrelevant information interference. This integrated strategy greatly improves the recognition and segmentation accuracy of rice leaf disease detection, ensuring the model’s robustness and efficiency in complex backgrounds. The CRM structure is shown in Fig. 3E, and the SFAM experiments are described in the “Effectiveness of the SFAM” section.

#### Annealing-integrated sparrow optimization algorithm

In the task of semantic segmentation of diseased rice leaves, the fuzzy boundary near the diseased spot has a large impact on the segmentation network. Selecting efficient optimizers such as SGD [38] and Adam [39] can accelerate model convergence through adaptive learning rates. SGD uses random samples to update parameters and can include momentum to speed up learning. Adam achieves finer learning rate tuning by calculating EMAs of the gradient, and squared EMAs for normalizing and bias-correcting the gradient. However, fuzzy edges lead to weak gradient signals, and adaptive learning rate optimizers, such as Adam, have difficulty accurately estimating the gradient, which affects learning and generalization. In contrast, AdamW [40] adds decoupling weights and parameter updates to Adam to provide more effective regularization, which is suitable for processing complex images, but its exploratory ability and update strategy are slightly insufficient. Therefore, based on AdamW, we introduce the sparrow search algorithm [41] and simulated annealing algorithm [42] and propose a new optimizer algorithm, the AISOA, to better capture fuzzy diseased leaf features, stabilize training, and improve segmentation accuracy.

The optimization parameter process of the AISOA consists of the following steps. First, the initial parameters of the optimizer are set, such as the learning rate lr, the momentum term beats, the smoothing term eps, the weight decay weight_decay, the annealing rate anneal_rate, the updating interval update_interval, and the perturbation parameter sparrow_factor. Next, the parameters are updated with AdamW, the exponential average of the first-order moments (mean) and second-order moments (variance) of their gradients are computed, bias corrections are applied, and the weights are updated based on these computed values. This process can be expressed in the following equation:

$$\left\{\begin{array}{c} m_{t}=\beta_{1}\cdot m_{t-1}+\left(1-\beta_{1}\right)\cdot g_{t}\\ v_{t}=\beta_{2}\cdot v_{t-1}+\left(1-\beta_{2}\right)\cdot g_{t}^{2}\\ {\hat{m}}_{t}=\frac{m_{t}}{1-\beta_{1}^{t}}\\ {\hat{v}}_{t}=\frac{v_{t}}{1-\beta_{2}^{t}}\\ \theta_{t}=\theta_{t-1}-\eta_{t}{\lambda \theta }_{t-1}-\eta_{t}\frac{m_{t}}{\sqrt{{\hat{v}}_{t}}+\in } \end{array}\right.$$ (16)

where *m_t_* and *v_t_* are the EMAs of the first- and second-order moments at moment *t*, respectively; *g_t_* is the scale at moment *t*; and *β*_1_ and *β*_2_ are the momentum decay rates. ${\hat{m}}_{t}$ and ${\hat{v}}_{t}$ are the bias-corrected moments, *θ_t_* is the weight at step *t*, *η_t_* is the learning rate at step *t*, and λ is the coefficient of the weight decay. ε is a constant to prevent division by zero errors.

The conventional AdamW optimizer has no additional mechanism to facilitate exploration of the parameter space and is prone to falling into local optima, leading to reduced generalizability. Therefore, we choose to introduce the sparrow optimization algorithm (SOA), which is more capable and efficient in exploration, by introducing its key idea, the introduction of randomness to simulate the natural behavior of sparrows, into the AdamW optimizer. This helps to increase the diversity of the solution space and prevents the algorithm from converging prematurely to a local optimum. The optimizer’s performance in complex optimization environments is enhanced, such as in situations with disease objects with fuzzy edges, and its ability to generalize is also improved. The logic of the joined SOA is that through the introduction of the update_interval parameter mentioned above, we apply a random perturbation to the model parameter variable after each update_interval step, and this periodic perturbation is achieved by multiplying the sparrow_factor by a Gaussian random number, which has the following mathematical expression:

θt+1=θt+η⋅sparrowf_actort⋅N1−I (17)

where *N* (0, *I*) is a Gaussian random vector with mean 0, and the covariance matrix is the unit matrix, and η denotes the learning rate.

Finally, considering that the increase in computation caused by the continuous application of the SOA throughout the training process is likely to affect the efficiency of model training, we integrate the annealing mechanism, the core idea of which is borrowed from the simulated annealing process in physics, where annealing is a heat treatment process that reduces internal defects in a material by gradually lowering the temperature of the material to achieve a more stable state. In the AISOA, we use the annealing mechanism to gradually adjust the optimization parameters to improve the efficiency and quality of the search for the optimal solution. Our annealing mechanism achieves this by gradually decreasing the value of the sparrow_factor. A greater sparrow_factor allows for a greater magnitude of stochastic perturbations in the early stages of training, facilitating extensive exploration of the parameter space and helping the optimizer jump out of the locally optimal solution, whereas as training progresses, the sparrow_factor is gradually reduced to reduce the magnitude of the stochastic perturbations, thereby reducing the intensity of exploration and allowing the optimizer to tune the parameters more carefully to find a more accurate optimal solution. The mathematical expression is as follows:

sparrowf-actort+1=sparrowf-actort⋅1−annealrate-⋅globalstep- (18)

where global_step is the global step used to track the number of times the optimizer calls the method.

By employing the AISOA optimizer, the model performs much better on the task of segmenting the fuzzy edges of rice leaves. AISOA combines the annealing mechanism and periodic perturbation, which not only enhances the model's ability to avoid falling into local optimums but also helps to identify and learn difficult features such as fuzzy edges. This optimization strategy motivates the model to capture edge information more accurately in complex backgrounds, improving the accuracy and robustness of the segmentations. In conclusion, the use of the AISOA optimizer significantly optimizes rice leaf disease segmentation, especially for regions with unclear edges. Experiments on the AISOA are described in the “Effectiveness of the AISOA” section.

## Results and Analysis

### Experimental environment

To prevent different experimental environments from affecting the results, all the experiments in this paper were run in the same hardware and software environment. The main hardware devices used in this experiment were an NVIDIA GeForce RTX A5000 (24 GB) and a 15 vCPU AMD EPYC 7543 32-Core Processor. However, the versions of Python, CUDA, and CUDNN did not affect the results of the experiment but needed to be compatible with the software and hardware. We implemented AISOA-SSformer on PyTorch 1.10.0.

### Experimental indicators

To evaluate the segmentation results, we used the MIoU, Dice coefficient [43], precision, and recall as evaluation metrics. The Dice coefficient is a measure of the similarity between the true value and the predicted output in the segmentation results. The MIoU measures the degree of overlap between the spot areas predicted by the model and the actual spot areas. Precision indicates how many of the areas predicted by the model to be spots are correct. Recall represents the proportion of the spot areas that the model can correctly identify to all the actual spot areas. The formulas for the above 4 metrics are as follows:

$$\text{MIoU}=\frac{1}{N}\sum_{i=1}^{N}\frac{{\mathrm{TP}}_{i}}{{\mathrm{TP}}_{i}+{\mathrm{FP}}_{i}+{\mathrm{FN}}_{i}}$$ (19)

$$\text{Dice coefficient}=\frac{\mathrm{TP}\cap \text{Label}}{\mathrm{TP}\cup \text{Label}}$$ (20)

$$\text{Precision}=\frac{\mathrm{TP}}{\mathrm{TP}+\mathrm{FP}}$$ (21)

$$\text{Recall}=\frac{\mathrm{TP}}{\mathrm{TP}+\mathrm{FN}}$$ (22)

where TP, FP, and FN are true-positive, false-positive, and false-negative measurements, respectively.

GFLOPs, or Giga Floating-Point Operations per Second, are used to denote the computational complexity of a model or algorithm in processing a task. They measure the number of floating-point operations performed in each second. To calculate GFLOPs, you count the total number of floating-point operations required to complete a task and then divide by one billion (since “Giga” denotes a billion). 

### Hyperparameter settings

In the Segformer source code, the recommended number of iterations for the Segformer_b5 model is 1.6 × 10^5^. However, our benchmark experiments used the Segformer_b0 model, which is smaller and has fewer parameters than the Segformer_b5 model. Therefore, we conducted an 1.6 × 10^5^ iteration experiment and observed its loss curve and found that when the number of model fitting iterations was approximately 8 × 10^4^, the loss value was similar to that of 1.6 × 10^5^ iterations, which was approximately 0.018. Therefore, we decided to set the number of iterations to 8 × 10^4^ to improve the model training efficiency. The other training hyperparameters are shown below:

where the training set contains 1,604 images, the test set contains 401 images, and the size of the input images is 256 × 256. We used the AISOA optimizer with the learning rate set to 1 × 10^−3^ and the weight decay factor set to 1 × 10^−2^ with a polynomial decay strategy for learning rate tuning. The batch_size was set to 2, and the baseline model was Segformer_b0. The number of training iterations was 8 × 10^4^.

### Module effectiveness experiments

#### Effectiveness of SGUP

In this paper, we use the SGUP instead of the traditional MLP to enhance the stability of the model in the face of irregular features. In the “Sparse global-update perceptron” section, we mentioned that a sliding window of size “*n*” is designed to smoothly update the parameters by considering the parameter values within this sliding window to reduce the parameter variations caused by word iterations. In this experiment, we compare the effect of the SGUP under different values of *n*. The experimental results are shown in scheme 1 in Table 2.

**Table 2.** Results of the AISOA-SSformer module experiment

Group	Method	MIoU	Dice coefficient	Recall
①	MLP	0.782	0.729	0.619
	SGUP (*n* = 10)	0.772	0.714	0.596
	SGUP (*n* = 20)	0.793	0.743	0.642
	SGUP (*n* = 30)	0.752	0.681	0.531
	SGUP (*n* = 40)	0.787	0.708	0.628
	SGUP (*n* = 50)	0.762	0.698	0.562
②	Without attention	0.782	0.729	0.619
	Without SRM and CRM	0.787	0.715	0.663
	With SRM	0.795	0.751	0.751
	With CRM	0.796	0.752	0.752
	SFAM	0.809	0.771	0.716
③	Without attention	0.782	0.729	0.619
	SE	0.772	0.715	0.701
	CBAM	0.788	0.738	0.618
	TA	0.792	0.761	0.657
	CA	0.786	0.737	0.629
	SFAM	0.809	0.771	0.716
④	AdamW	0.782	0.729	0.619
	Adam	0.775	0.719	0.636
	Lion	0.789	0.732	0.651
	RAdam	0.763	0.701	0.639
	RMSprop	0.744	0.668	0.565
	Only SOA	0.781	0.729	0.655
	AISOA	0.794	0.749	0.656

From the experimental results, it can be seen that when the value of n is small or large, the effect on Segformer is small or even negative, which means that when the value of n is too large, the model needs to store more historical parameters, which increases the memory demand, and the calculation of the weighted average involves more data points, which increases the data overhead, resulting in a sluggish response. When the value of *n* is too small, the model will be more sensitive to the transformations at each iteration when the parameters are updated, which leads to oscillations during the training process and reduces the generalization ability of the model. Therefore, we choose an intermediate value of *n* = 20 as the size of the sliding window in the SGUP.

#### Effectiveness of the SFAM

To demonstrate the effectiveness and superiority of SRM and CRM for the network, we conducted experiments with and without the addition of SRM and CRM alone and without the addition of the 2 modules, and the results of the experiments are shown in scheme 2 of Table 2.

The experimental results show that attention has little effect on the network’s enhancement when no SRM or CRM is added, while the performance of the network is improved to a certain extent after SRM and CRM are added separately, and the best effect is achieved when both are added, which indicates that the gating mechanism and feature reconstruction capability of SRM and the channel compression and change mechanism of CRM are effective, which can make the network process and utilize the feature information more efficiently.

To test the overall performance of the SFAM, we introduced other attention mechanisms, such as SE, CBAM [44], triplet attention (TA) [45], ECA [46], and CA, at the same locations in the network. The experimental results are shown in scheme 3 of Table 2.

The experimental results show that, excluding SE, the remaining attention can filter the interference information and improve the segmentation accuracy to some extent, but the effect is not significant. This is because SE mainly focuses on channel attention, which ignores the importance of spatial information while enhancing channel features, which is particularly important for image segmentation, so the addition of SE leads to a reduction in segmentation accuracy. CBAM, although it considers spatial attention, is still deficient in capturing contextual information. The TA focuses on applying attention in different dimensions but does not integrate this information well, so it is less effective. The SFAM is optimal for all 3 key metrics, improving the model’s segmentation accuracy for rice leaf disease images with complex backgrounds.

**Fig. 4.**
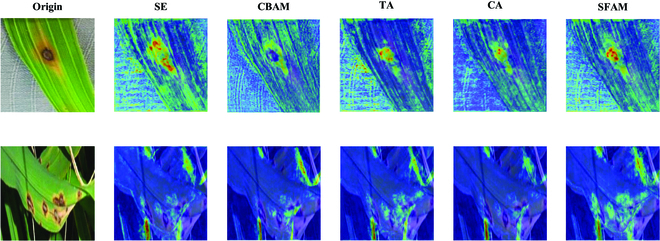
Comparison of attention maps for different types of attention.

To demonstrate the effectiveness of the SFAM in semantic segmentation of rice leaf disease more intuitively, we compared the attention maps of the above methods, as shown in Fig. 4.

By comparing attention maps, it is clearly observable that SFAM exhibits superior capabilities in the identification and precise localization of disease features on rice leaves. It demonstrates exceptional precision and coverage across various background conditions, whether simple or complex (such as those with shadows and weeds). In simple backgrounds, all the attention mechanisms can focus on the disease features, but SFAM’s focal points are more accurate. With more complex backgrounds, the performance advantage of the SFAM becomes even more pronounced. It accurately highlights diseased areas while reducing attention to background noise. Such precision is crucial for practical applications because it ensures the algorithm’s reliability and robustness in real-world scenarios. Overall, the SFAM had the highest comprehensive indicator scores and had stronger localization capabilities for diseased areas, proving its suitability for the recognition and segmentation of rice leaf diseases.

#### Effectiveness of the AISOA

**Fig. 5.**
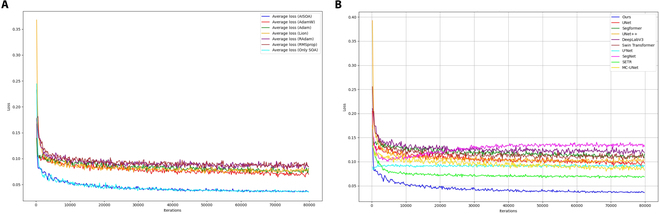
(A and B) Verification loss curves for different optimizers and networks.

In this paper, we incorporate the Sparrow algorithm and annealing mechanism based on AdamW to improve the model’s segmentation ability and robustness to fuzzy edges. We conduct experiments on several advanced optimizer algorithms (Adam, Lion [47], RAdam [48], and RMSprop [49]) under the same experimental configuration and compare them with experiments that only add the SOA. The loss function curves obtained during the validation process and the experimental results are shown in scheme 4 of Table 2 and Fig. 5A, respectively.

The experimental results show that compared to other optimizers, the AISOA exhibits better performance on our homemade dataset. First, it has a significantly lower loss curve, which indicates that AISOA has better convergence performance on the dataset. Especially in the early stage of training, the loss of the AISOA decreases rapidly, which means that it can learn faster and find the optimal solution quickly. In contrast, when comparing the optimizers after adding only SOA, although there is little difference in the loss curves, due to the lack of an annealing mechanism in SOA to downscale the search magnitude, the model has excessive training computations, which leads to a lower segmentation performance. AISOA also leads in key performance metrics, achieving the highest scores in both the MIoU and Dice coefficient, with values of 0.794 and 0.749, respectively. This further demonstrates its accuracy in image segmentation tasks. Overall, the AISOA effectively suppresses fuzzy edge features through its progressive stochastic search method, which ensures a stable convergence of the model while obtaining the lowest loss curve. This approach improves the segmentation accuracy and enables the AISOA to outperform current popular optimizer algorithms in terms of performance.

### Ablation experiments

To verify the effectiveness of the proposed Segformer-based approach, we conducted ablation experiments on AISOA-SSformer, and the results of the experiments are shown in Table 3. We integrate SGUP, AISOA, and SFAM sequentially using the control variable method and conduct 8 sets of ablation experiments by combining these 3 AISOA-SSformer modules. The experimental results show that our proposed SFAM can significantly improve the segmentation performance of the network, which can improve the MIoU by 3.45% and the Dice coefficient by 5.79% on average under different conditions, which proves that the SFAM is able to improve the segmentation accuracy of diseased rice leaves by performing accurate feature extractions on the feature maps in both the length and width dimensions simultaneously in both the channel and space. In addition, we optimized the network using SGUP and AISOA, which are still effective, although their MIoU and Dice coefficient improvements are relatively small, at 1.41% and 1.92% and 1.53% and 2.75%, respectively. In conclusion, each module of AISOA-SSformer positively affects the MIoU and Dice coefficients of the model segmentation, which confirms that our proposed SGUP, AISOA, and SFAM are effective.

**Table 3.** Comparison of ablation results

	SGUP	SFAM	AISOA	MIoU	Dice coefficient
Segformer				0.782	0.729
	√			0.793	0.743
		√		0.809	0.771
			√	0.794	0.749
	√	√		0.824	0.771
	√		√	0.813	0.776
		√	√	0.817	0.793
	√	√	√	0.831	0.803

### Comparison experiments with other networks

**Fig. 6.**
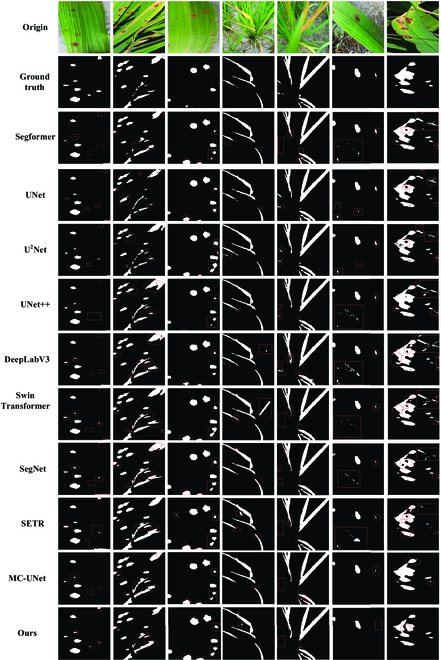
Examples of segmentation results for different networks.

To further analyze the performance of AISOA-SSformer, we conducted comparative experiments with several classical and current state-of-the-art target detection methods on the same test environment and test set and plotted the loss plots of all the network training processes. The experimental results are shown in Table 4 and Fig. 6. We circle the locations of the incorrectly segmented and missing (or extra) segments for each network, where the oval boxes are incorrectly segmented segments and the rectangular boxes are missing (or extra) segments. The loss curve comparison plots are shown in Fig. 5B.

**Table 4.** Comparison of the performances of the AISOA-SSformer and other networks

Model	MIoU	Dice coefficient	Recall	Precision	Parameters	GFLOPs
Segformer [18]	0.782	0.729	0.619	0.889	14.15 million	3.38G
UNet [12]	0.768	0.707	0.599	0.961	24.89 million	31.12G
U^2^Net [54]	0.773	0.715	0.621	0.842	167.71 million	37.65G
UNET++ [55]	0.778	0.722	0.619	0.868	34.96 million	34.87G
DeepLabV3 [56]	0.802	0.761	0.696	0.838	42.07 million	2.49G
Swin Transformer [57]	0.812	0.776	0.721	0.841	107.89 million	4.37G
SegNet [58]	0.795	0.749	0.701	0.807	112.32 million	40.17G
SETR [59]	0.824	0.772	0.739	0.853	370.41 million	15.90G
MC-UNet [30]	0.803	0.769	0.704	0.845	6.67 million	18.00G
Ours	0.831	0.803	0.765	0.892	14.71 million	3.28G

Among the CNN-based [50] algorithms, UNet increases feature depth and fuses features in the encoder through a symmetric decoder-encoder structure but relies on localized regions, making it difficult to capture small changes in complex backgrounds. U^2^Net enhances detail fusions with a deeper nested U-shaped structure, but its multilayer structure increases computational complexity and reduces efficiency. UNet++ adds dense jump connections to UNet, lowering the computational complexity but still focuses on local features while ignoring the broader contextual information. SegNet uses encoder pooling indices for upsampling to capture spatial details but struggles with differentiating features in complex backgrounds. The MC-UNet model relies on local convolutional operations for feature extractions and often omits long-distance correlations between rice disease pixels.

For Transformer or hybrid architectures, DeepLabV3 uses hollow convolution and ASPP modules to emphasize the multiscale information, but this can cause the model to overlook necessary details. The Swin Transformer incorporates self-attention and a hierarchical structure but lacks sensitivity in the fine processing of small regions. SETR combines global self-attention with traditional convolutional networks and excels in handling large-area features but has too many parameters and insufficient precision for small-range features. The experimental results show that our proposed AISOA-SSformer outperforms traditional and popular semantic segmentation algorithms in terms of the MIoU, Dice coefficient, and recall, is second only to UNet in terms of precision, is slightly more complex than Segformer, and has the lowest loss value, demonstrating its efficiency and accuracy in extracting the complex features of rice leaf diseases.

Compared to the other models in the table, AISOA-SSformer is the most suitable model for rice leaf disease segmentation. We speculate that our AISOA-SSformer outperforms the other models for the following reasons: (a) AISOA-SSformer is an improvement on Segformer. This study designed a novel hierarchical Transformer, and since rice disease areas may behave differently at different scales, this hierarchical approach helps the model identify and segment different types of diseases more accurately and improves the accuracy of the model. (b) The adoption of the SGUP instead of the MLP in AISOA-SSformer improves the model’s smoothing inference and updating of global parameters, and accurately captures and retains key features in rice leaf disease segmentations. (c) The introduction of the SFAM greatly enhances the ability of the network to fuse features at different levels. This optimization significantly improves the model’s efficiency in perceiving and recognizing foreground features, which is especially critical for accurately distinguishing small differences between rice and complex backgrounds. As a result, the model exhibits higher accuracy in recognizing the boundary between rice and complex backgrounds. (d) Substituting the AISOA for AdamW in the original network avoids falling into a local optimum during model training and helps the model learn difficult-to-capture features more efficiently. The ability to explore a wider parameter space is crucial for improving model performance when dealing with disease images with blurred edges. (e) The homemade dataset in this paper eliminates some blurred and lower-quality images, and its images are clear and favorable for model training.

### Discussion

**Fig. 7.**
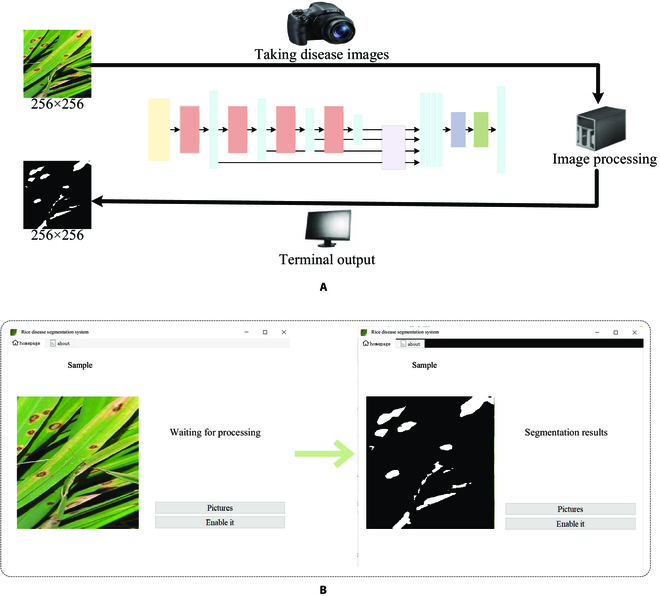
(A and B) Schematic diagram of the disease segmentation system and system demonstration example.

To test the effectiveness of the AISOA-SSformer model in rice leaf disease segmentation, we built a rice leaf disease segmentation system. Figure 7 shows (A) an architectural diagram of the system and (B) a running example of the executable. As shown in the figure, we created a Pyqt interface to act as our system. After deployment, we uploaded the rice disease images captured in the field to the local host, entered the system, and clicked “Enable it” to start the segmentation, and the segmentation results were directly displayed on the system interface to help people analyze and evaluate the rice diseases in a targeted way.

**Fig. 8.**
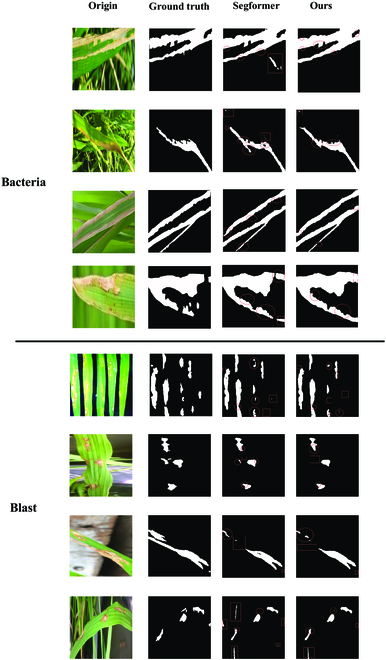
Visual comparison of segmentation of unknown datasets under 200 training samples.

To verify the generalization ability of AISOA-SSformer and the segmentation effect on different disease datasets, we further expanded the experimental scope. We continued to expand the dataset and traveled to Yue yang, Hunan Province, to take field photos of rice leaf diseases. With the help of experts from the Institute of Plant Protection, Chinese Academy of Agricultural Sciences (CAAS), and after careful screening and identification of disease pathogens using polymerase chain reaction (PCR), we selected 2 common rice leaf diseases, bacterial leaf blight of rice (bacteria) and rice blast (blast), each had 300 available images, for generalizability experiments. In addition, to simulate real farmland with less disease image data, we conducted experiments without adding any training samples, adding 100 training samples, and adding 200 training samples. The metrics for these 3 experiments are shown in Table 5, where the first part is without training samples, the second part is with the addition of 100 training samples, and the third part is with the addition of 200 training samples. The visualization results of the experiment in which 200 training samples were added are shown in Fig. 8.

**Table 5.** Comparison of segmentation metrics for unknown datasets with different training samples

Group	Disease type	Method	MIoU	Dice coefficient	Recall	Precision
①	Bacteria	Ours	0.628	0.518	0.392	0.754
		Segformer	0.553	0.345	0.222	0.781
	Blast	Ours	0.681	0.577	0.449	0.771
		Segformer	0.568	0.333	0.214	0.744
②	Bacteria	Ours	0.743	0.737	0.635	0.859
		Segformer	0.714	0.678	0.605	0.825
	Blast	Ours	0.722	0.654	0.538	0.833
		Segformer	0.648	0.531	0.479	0.694
③	Bacteria	Ours	0.812	0.801	0.746	0.872
		Segformer	0.782	0.763	0.665	0.895
	Blast	Ours	0.786	0.739	0.617	0.834
		Segformer	0.742	0.674	0.538	0.833

The results show that AISOA-SSformer outperforms Segformer in segmenting unknown types of rice diseases (e.g., bacteria and blast) with higher segmentation metrics. This finding highlights the significant advantage of AISOA-SSformer in terms of its generalizability. In particular, in the case of complex and intrusive backgrounds (e.g., human interference), the performance of Segformer is only barely passable, whereas AISOA-SSformer significantly outperforms Segformer, demonstrating a stronger processing capability. With the addition of a small number of training samples, the metrics of AISOA-SSformer increase significantly, indicating that AISOA-SSformer is able to segment rice leaf diseases more accurately with the same dataset size. Specifically, after adding 100 training samples, the model performance improved significantly, showing a stronger learning ability and disease feature recognition. When the training samples are increased to 200, the performance is further improved, demonstrating superiority on small sample datasets. The addition of a small number of training samples significantly enhances the accuracy and robustness of the model compared to the case where no training samples are added. In summary, the experimental results demonstrate the superior performance of AISOA-SSformer in rice leaf disease segmentation tasks, especially in the case of a small number of training samples, which has potential for wide application. However, it still has slightly lower metrics in predicting unknown disease categories compared to the 2 trained disease categories, and we also found some cases of missed segmentation and incorrect segmentation, which suggests that our network model is more suitable for segmenting those rice leaf disease images that have been included in the training data. In addition, although the model is more capable of handling complex backgrounds, the performance may still be limited under extreme conditions, such as when there are drastic changes in light, overlapping leaves, or many occlusions. These conditions are more common in natural environments, so improving the robustness of the model in the face of these challenges is a focus of future research.

Given our ultimate goal of applying the network to practical rice disease analyses, we anticipate that the images we will need to process will cover a wider variety of diseases and be accompanied by more complex backgrounds. To address these challenges, we plan to adopt the following 3 main strategies in our future work. First, we will expand the training dataset to cover a broader range of disease types and background conditions. Second, we will explore the possibility of using multistage segmentation models to improve the handling of diverse diseases and complex backgrounds. Finally, we will lightweight and quantitatively deploy the model on edge devices using methods such as knowledge distillation to ensure efficient and accurate identification and segmentation of rice leaf diseases in practical deployments. By combining these measures and optimizations, we hope to develop a more robust and reliable automatic rice disease identification and segmentation system that can not only accurately process existing and unknown disease types but also adapt to various complex environmental conditions, thereby providing efficient technical support for agricultural production.

## Conclusion

Rice leaf disease and pest segmentation is an important technology for precision agriculture and crop health management that provides accurate information on rice leaf diseases and helps farmers assess disease severity and progress. This technology helps farmers make more effective decisions by assessing disease severity and progression in a timely manner. Through this approach, crop management efficiency and yield can be significantly improved while reducing the use of chemical pesticides and promoting sustainable agriculture. However, rice leaf pest and disease segmentation also faces some challenges, such as irregular spots, blurred edge textures, and cluttered and complex background elements. To solve these problems, a new Transformer segmentation network, AISOA-SSformer, is proposed in this paper, which achieves better results in rice leaf pest and disease segmentation.

a. Ablation experiments showed that SGUP, SFAM, and AISOA were more effective in rice leaf pest and disease segmentation, with +1.41%, +3.45%, and +1.53% MIoUs, respectively. Under the same experimental setup, compared with Segformer, AISOA-SSformer improved the MIoU and Dice coefficient by 6.27% and 10.14%, respectively.

b. Compared with the current mainstream segmentation algorithm pairs, the MIoU of AISOA-SSformer is 83.1%, the DICE is 80.3%, the recall is 76.5%, and the size of the model is only 14.71 million. The method shows excellent performance, not only in terms of segmentation accuracy but also in the handling of complex backgrounds and irregular lesions. AISOA-SSformer has excellent performance and has wide application potential in the field of agricultural disease management and intelligent disease diagnosis.

In this paper, an innovative rice leaf pest and disease segmentation method (AISOA-SSformer) is proposed. This method significantly enhances the ability to recognize rice leaf pest and disease features by effectively extracting and fusing features at different scales and levels, thereby improving the consistency of intraclass predictions. According to a series of experiments, AISOA-SSformer performs well on several key performance metrics, including the MIoU, Dice coefficient, and recall. These results not only show the superior performance of AISOA-SSformer in pest and disease segmentation but also highlight its excellent generalizability. The successful application of this method is important for precision agriculture and crop health management. By accurately segmenting and identifying pests and diseases on rice leaves, AISOA-SSformer can provide farmers and agricultural experts with critical information to help them assess and manage crop diseases more effectively, thus realizing practical application value in multiple ways. First, AISOA-SSformer can significantly reduce the use of pesticides. By accurately segmenting pests and diseases, farmers can target pest control, avoiding the blind and excessive use of pesticides, thus protecting the environment and reducing pesticide residues. Second, the method improves crop yields, preventing the spread of diseases and pests by identifying and treating pest and disease problems in a timely manner, guaranteeing healthy crop growth, and ultimately improving the yield and quality of rice. In the future, we plan to apply AISOA-SSformer to a wider range of agricultural tasks, including pest and disease segmentation in other crops. In addition, we will explore the adoption of more advanced deployment technologies, such as knowledge distillation, to further improve the processing efficiency of AISOA-SSformer and expand its application scenarios so that it can play a greater role in actual agricultural production. Through these efforts, we expect AISOA-SSformer to make greater contributions to smart agriculture and sustainable development.

## Data Availability

The datasets and code used in this study have been posted on the website https://github.com/ZhouGuoXiong/ Rice-Leaf-Disease-Segmentation-Dataset-Code.
